# Work Climate and nurses’ mental health: an observational study in an acute hospital ward

**DOI:** 10.1192/j.eurpsy.2025.1839

**Published:** 2025-08-26

**Authors:** C. Laranjeira, M. Dixe, A. Querido

**Affiliations:** 1 School of Health Sciences; 2 ciTechCare, Polytechnic Institute of Leiria, Leiria, Portugal

## Abstract

**Introduction:**

Since the COVID-19 pandemic, the attention to mental health at work has increased. According to recent WHO reports, 12 billion working days are lost every year to depression and anxiety. The working conditions, particularly the organizational climate, can impact nurses’ well-being and mental health. Specifically, in Portuguese nurses, little is known about the relationship between work climate and mental health.

**Objectives:**

To characterize the nurses’ lifestyle and mental health and correlate the organizational climate to nurses’ mental health.

**Methods:**

A cross-sectional correlational study was conducted in a Medical-surgical ward in a Portuguese acute hospital. A convenience sample was recruited, excluding the nurses with long-term sick leave. An e-survey was sent to the nurses by the head nurse via institutional emails after the approval of the Ethics Committee. The nurses were asked to fill in sociodemographic, professional, anthropometric and lifestyle inquiry, Mental Health Inventory (MHI– 5) and Work Climate Questionnaire (WCQ). Data was analyzed using SPSS 29.0.

**Results:**

46 Portuguese nurses participated in the study. Most women (87%), aged between 26 and 64 years, have worked for 18 years (±8.4). Most nurses consider BMI median=25.6 (±5.4), 75% exercise regularly, 93% are non-smokers, and 67% consume alcohol occasionally. Most nurses rated their sleep as poor quality. Nurses considered they have good health (43.5%) in general and evidenced reasonable mental health (MHI Mean=19.8± 3.9). Male nurses experienced higher mental well-being than females. As for the level of organizational climate, it was found that, on average, scores are close to 4 (neither agree nor disagree). There was a positive correlation between organizational climate and nurses’ mental health.

**Conclusions:**

This study evidenced a neutral perception of confidence and experience of autonomy of nurses working in an acute hospital in Portugal. There is a need to deepen the knowledge of the level of autonomy experienced by nurses in their work in larger samples and different contexts. The results indicate the need to study this correlation in different settings to explore its effect and impact on nurses’ mental health and patient quality of care.

**Disclosure of Interest:**

None Declared

